# Analysis of the Changes and Possible Reasons in Aberrations before and after Surgery in Patients with Concomitant Exotropia

**DOI:** 10.1155/2022/5207553

**Published:** 2022-09-05

**Authors:** Xixi Zhang, Jiajia Zhou, Haoliang Li, Jiajun Lu, Shu Liang

**Affiliations:** Department of Ophthalmology, Affiliated Hospital of Nantong University, Medical School of Nantong University, Nantong 226001, China

## Abstract

**Objective:**

The objective is to observe the changes in aberrations before and after surgery in patients with common horizontal strabismus and to analyze the possible reasons for the changes.

**Methods:**

Forty eyes of 40 cases with concomitant exotropia who underwent strabismus correction at the Ophthalmology Department of Nantong University Hospital from October 2020 to July 2021 were included in this study, all of whom underwent unilateral lateral rectus recession combined with a medial rectus resection in the same eye. Aberration parameters were measured 1 day before surgery and 1 week, 1 month, 3 months, and 6 months after surgery. Differences in the indicators at each time period were compared by analysis of variance (ANOVA) of repeated measures data for a single factor, and data were analyzed using SPSS 25.0 statistical application software.

**Results:**

5 mm pupil diameter: the preoperative and postoperative RMS of total aberration showed statistically significant difference (*P* < 0.01). Postoperation test (Bonferroni method) and preoperative comparison at each period after surgery showed statistically significant differences between 6 months after surgery (*P*=0.002) and preoperative comparison. The preoperative and postoperative comparison of RMS in LOAs was statistically significant (*P* < 0.01); postoperative test (Bonferroni method) and preoperative comparison showed that there were statistically significant differences between 1 week (*P*=0.033) and 6 months (*P*=0.002) after operation. The difference of RMS of defocus before and after operation was statistically significant (*P* < 0.01); postoperation test (Bonferroni method) and preoperative comparison showed that there was statistically significant difference between 6 months after operation (*P*=0.007) and preoperative comparison. There was statistically significant difference in preoperative and postoperative RMS of HOAs (*P*=0.013). Postoperative test (Bonferroni method) and preoperative comparison showed that there was statistically significant difference 6 months after surgery (*P*=0.03). The RMS of secondary astigmatism showed a statistically significant difference before and after operation (*P*=0.001), and the postoperation test (Bonferroni method) showed a statistically significant difference 6 months after operation (*P*=0.002). In 5 mm pupil diameter, the preoperative and postoperative RMS of total aberration showed statistically significant difference (*P* < 0.01), postoperative test (Bonferroni method) was used to compare each period after surgery with that before surgery, and there were statistically significant differences between 1 week after surgery (*P*=0.034), 3 months after surgery (*P*=0.033), and 6 months after surgery (*P*=0.003). The preoperative and postoperative comparison of RMS in LOAs was statistically significant (*P* < 0.01), postoperative test (Bonferroni method) was used to compare each period after surgery with that before surgery, and there were statistically significant differences between 1 week after surgery (*P*=0.04), 3 months after surgery (*P*=0.034), and 6 months after surgery (*P*=0.004). The difference of RMS of defocus before and after surgery was statistically significant (*P*=0.002), and the comparison between postoperation test (Bonferroni method) and preoperation showed that the difference was statistically significant 6 months after surgery (*P*=0.027). The RMS of astigmatism showed statistically significant difference before and after operation (*P*=0.002), and the postoperation test (Bonferroni method) showed statistically significant difference between 6 months after operation (*P*=0.009) and before operation.

**Conclusion:**

We found that horizontal rectus surgery had a transient effect on LOAs and almost no effect on HOAs. Long-term follow-up is recommended after strabismus surgery to observe eye position and binocular visual function. Because of the high prevalence of strabismus in adolescents, long-term observation of the eye axis and aberration is recommended.

## 1. Introduction

Recent studies report that the global prevalence of strabismus is as high as 1.93%, with a worldwide estimated pooled prevalence of 1.78% for those under 20 years of age and 3.29% for those over 20 years of age [1–3]. Surgery is a common treatment for strabismus. It not only corrects the eye position and thus improves the appearance, but also helps patients with strabismus to reestablish visual function in both eyes [4].

The human eye includes low order aberrations (LOAs) and high order aberrations (HOAs). LOAs refer to the 1st and 2nd order aberrations, which represent traditional refractive problems such as defocus and astigmatism, mainly affecting vision, and can be corrected by spherical and cylindrical lenses. Aberrations that affect the image quality of the retina and cannot be corrected with cylindrical lenses are called HOAs, which are 3rd and higher-order aberrations, including spherical and coma aberrations. The main higher-order aberrations that degrade visual quality are coma, spherical aberration, trefoil, and secondary astigmatism, which cause ghosting, glare, asterism, and mixed focus.

Although HOAs affect visual imaging quality, previous studies have mainly dealt only with refractive errors after strabismus surgery, not HOAs. There are reports that surgery can cause transient myopic drift or lead to astigmatism and axial changes in patients, but the effects are transient and reversible [5–9], while others believe that surgery may cause long-term irreversible refractive changes [10]. Some investigators have also not observed differences in equivalent spherical lenses in strabismus surgery [11, 12]. Some strabismus surgery patients complain of blurred vision and poor visual quality after surgery but there are no significant changes in visual acuity and refractive status on examination, and we speculate that such patients may be affected by postoperative changes in HOAs. However, there is still little information in the literature regarding postoperative measurement of aberration changes in strabismus. Therefore, the purpose of this study was to observe the changes in aberrations before and after surgery in patients with common horizontal strabismus and to analyze the possible reasons for the changes.

## 2. Methods

### 2.1. Subject Selection

This was a retrospective study. Forty eyes of 40 cases with concomitant exotropia who underwent strabismus correction at the Ophthalmology Department of Nantong University Hospital from October 2020 to July 2021 were included in this study, all of whom underwent unilateral lateral rectus recession combined with a medial rectus resection in the same eye, age 5 to 54 years, median age 11 (7, 22), and deviation: −40^△^ to −110^△^. All subjects voluntarily cooperated and signed the informed consent form after being informed of the purpose and potential risks and contents. All patients were voluntarily included in the trial.

#### 2.1.1. Inclusion Criteria

1. Age ≥5 years
2. First strabismus surgery
3. No history of other ocular and systemic diseases, no amblyopia, no obvious ocular motility disorders or vertical strabismus, no A-V sign, and no obvious nystagmus
4. All patients were able to cooperate with dilated pupils and other ophthalmologic examinations and could be reviewed regularly

#### 2.1.2. Exclusion Criteria

1. History of ocular trauma, surgery, endogenous eye disease, and family history of eye disease
2. Incomplete or missing postoperative follow-up data

### 2.2. Ophthalmic Examinations

Prior to surgery, all patients underwent a detailed ophthalmic evaluation. The corneal, lens, and fundus status were evaluated using a slit lamp (IEC 601–1, TOPCON, Japan). The angle of deviation was determined mainly by alternating prism coverage tests at distance 6 m and nearby 33 cm.

### 2.3. Surgery

All patients undergo exotropia surgery under the microscope by a highly experienced chief ophthalmologist. Each patient was treated with levofloxacin eye drops 3 times daily for 3 days prior to surgery. The surgical procedures were as follows: ophthalmic routine disinfection towel, eyelid opener to open the lid. The conjunctival sac was rinsed with 0.5% povidone iodine, an arc-shaped incision was made near the fornix, the inner rectus muscle was hooked, and the surrounding tissues and abstinent ligaments were bluntly separated. Two pairs of entrapped sutures were made with 6–0 absorbable wires at the required distance from the end of the muscle, and the muscles were separated 1 mm before the sutures and sutured at the end of the muscle, the external rectus muscle was hooked, and the surrounding tissue and abstinence ligament were bluntly separated. Two pairs of snares were made with 6–0 absorbable suture 2 mm from the end of the muscle. The muscle was severed 1 mm before the suture, and the superficial sclera at the required distance from the end of the muscle was sewn, and the bulbar conjunctiva was sutured discontinuously. Anti-inflammatory eye drops were administered 2–3 weeks after surgery and reviewed regularly.

### 2.4. Aberration Analysis

All relevant aberration data were examined by the iTrace visual function analyzer. Wavefront analyses were performed by the iTrace visual function analyzer (iTrace, Tracey Technologies, USA) preoperatively and at 1 week, 1 month, 3 months, and 6 months postoperatively. The aberration of the patient's natural maximum pupil state was examined in the same darkroom condition. To reduce operator bias, measurements were repeated three times for each eye, and the image with the fewest rejection points was selected for statistical analysis.

The iTrace visual function analyzer represents the aberration in terms of a Zernike term: *Z*_*n*_^*m*^, where *n* is the order of the term and *m* is the frequency of the term. The root mean square (RMS) is calculated by the iTrace software from the Zernike coefficients to offset the effects due to different orientations. The root mean square is a useful quantification of the aberration as a whole or its individual components by applying the following equation:

(1) $$\begin{array}{c} RMS=\sqrt{\sum_{nm}{\left(Z_{n}^{m}\right)}^{2}}. \end{array}$$

**Figure 1:**
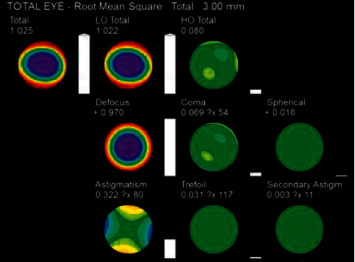
Whole-eye aberration at 3 mm pupil diameter.

**Figure 2:**
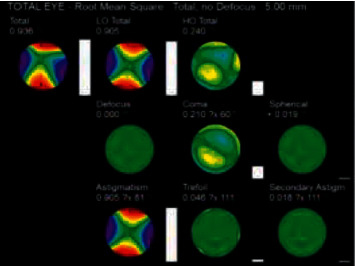
Whole-eye aberration at 5 mm pupil diameter.

The data collected in this study included the root mean square of whole-eye aberrations for pupil diameters of 3 mm and 5 mm (Figures 1 and 2), including total aberration, LOAs, defocus, astigmatism, HOAs, coma, spherical aberration, secondary astigmatism, and trefoil.

### 2.5. Statistical Analysis

All data were statistically analyzed by SPSS 25.0 software (SPSS Inc., Chicago, IL). The measured parameters for each study subject were expressed as mean ± standard deviation (SD). One-way repeated measures ANOVA was performed for preoperative and postoperative comparisons, and Bonferroni method was performed for postoperative comparisons at 1 week, 1 month, 3 months, 6 months and preoperatively. *P* < 0.05was considered statistically significant.

## 3. Result

A total of 40 patients (40 eyes) with concomitant exotropia were included in this study; 23 (57.5%) were male, with a median age of 10.5 (7, 22), range 5 to 54, and strabismus deviation of −40^△^ to −110^△^. The right eye was selected for observation in all patients, unilateral lateral rectus recession in one eye combined with a medial rectus resection. The maximum medial rectus resection was 5.5 mm and the minimum was 4 mm. Lateral rectus recession is 8 mm in maximum and 6 mm in minimum.

Table 1 shows a comparison of the RMS of preoperative and postoperative aberrations at 3 mm pupil diameter. The preoperative and postoperative RMS of total aberration showed statistically significant difference (*P* < 0.01). Postoperation test (Bonferroni method) and preoperative comparison at each period after surgery showed statistically significant differences between 6 months after surgery (*P*=0.002) and preoperative comparison, while there were no statistically significant differences between 1 week (*P*=0.065), 1 month (*P*=0.667), and 3 months (*P*=0.089) after surgery. The preoperative and postoperative comparison of RMS in LOAs was statistically significant (*P* < 0.01); postoperative test (Bonferroni method) and preoperative comparison showed that there were statistically significant differences between 1 week (*P*=0.033) and 6 months (*P*=0.002) after operation. There was no significant difference between 1 month (*P*=0.505) and 3 months (*P*=0.062) after surgery. The difference of RMS of defocus before and after operation was statistically significant (*P* < 0.01); postoperation test (Bonferroni method) and preoperative comparison showed that there was statistically significant difference between 6 months after operation (*P*=0.007) and preoperative comparison, while there was no statistically significant difference between 1 week (*P*=0.098), 1 month (*P*=1.0), and 3 months (*P*=0.111) after operation. There was statistically significant difference in preoperative and postoperative RMS of HOAs (*P*=0.013). Postoperative test (Bonferroni method) and preoperative comparison showed that there was statistically significant difference 6 months after surgery (*P*=0.03). There was no significant difference between 1 week (*P*=0.14), 1 month (*P*=0.808), and 3 months (*P*=1.0) after surgery. The RMS of secondary astigmatism showed a statistically significant difference before and after operation (*P*=0.001), and the postoperation test (Bonferroni method) showed a statistically significant difference 6 months after operation (*P*=0.002). There was no significant difference between 1 week (*P*=0.355), 1 month (*P*=0.282), and 3 months (*P*=1.0) after surgery. There was no significant difference in preoperative and postoperative RMS between astigmatism, coma, spherical aberration, and clover (*P* > 0.05).

The comparison of RMS of preoperative and postoperative aberrations at 5 mm pupil diameter is shown in Table 2. The preoperative and postoperative RMS of total aberration showed statistically significant difference (*P* < 0.01), postoperative test (Bonferroni method) was used to compare each period after surgery with that before surgery, and there were statistically significant differences between 1 week after surgery (*P*=0.034), 3 months after surgery (*P*=0.033), and 6 months after surgery (*P*=0.003). There was no significant difference between 1 month after surgery (*P*=1.0) and that before surgery. The preoperative and postoperative comparison of RMS in LOAs was statistically significant (*P* < 0.01), postoperative test (Bonferroni method) was used to compare each period after surgery with that before surgery, and there were statistically significant differences between 1 week after surgery (*P*=0.04), 3 months after surgery (*P*=0.034), and 6 months after surgery (*P*=0.004). There was no significant difference between 1 month after surgery (*P*=1.0) and that before surgery. The difference of RMS of defocus before and after surgery was statistically significant (*P*=0.002), and the comparison between postoperation test (Bonferroni method) and preoperation showed that the difference was statistically significant 6 months after surgery (*P*=0.027). There was no significant difference between 1 week (*P*=0.097), 1 month (*P*=1.0), and 3 months (*P*=0.06) after surgery. The RMS of astigmatism showed statistically significant difference before and after operation (*P*=0.002), and the postoperation test (Bonferroni method) showed statistically significant difference between 6 months after operation (*P*=0.009) and before operation. There was no significant difference between 1 week (*P*=0.191), 1 month (*P*=0.16), and 3 months (*P*=0.241) after surgery. There was no significant difference in preoperative and postoperative RMS between HOAs, coma, spherical aberration, secondary astigmatism, and trefoil (*P* > 0.05).

Table 3 shows the comparison of preoperative and postoperative eye axis lengths. The difference of axial length before and after operation was statistically significant (*P* < 0.01), postoperative test (Bonferroni method) was used to compare each period after surgery with that of before surgery, and it was found that there were statistically significant differences between 3 months after surgery (*P* = 0.026) and 6 months after surgery (*P* = 0.002), while there were no statistically significant differences between 1 week after surgery (*P* = 1.0) and 1 month after surgery (*P* = 1.0).

### 3.1. Discussion

Surgery is the main treatment for strabismus. Weakening and/or strengthening the horizontal rectus muscle to alter its direction of activity has been the treatment of choice for concomitant strabismus [13, 14]. A link has been established between strabismus surgery and postoperative changes in astigmatism, most of which are associated with changes in corneal curvature caused by changes in extraocular muscle tension transmitted through the sclera to the cornea. Other theories have been proposed for changes in astigmatism, including scleral wound healing [15], edema of the orbit and eyelids [10, 16], altered ciliary circulation, or changes in lens curvature [17]. Hainsworth [16]found that corneal curvature changed after strabismus. The change of muscle tension had little effect on the corneal curvature in the quadrant near the affected muscle but had a great effect on the entire surface of corneal curvature, and there were interactions and interdependence between various positions on the anterior surface of the cornea. Schworm [18] observed a brief change in postoperative corneal curvature, with flattening of the shortened cornea and steepening of the posterior cornea. At present, there is no unified conclusion about the change trend, duration, and whether there are different changes in refractive state and corneal curvature caused by strabismus correction. Most of the literature on changes in refraction after strabismus surgery shows that changes in the equivalent spherical lens, drift toward myopia, and an increase in astigmatism can be observed after strabismus surgery [5–9]. Some studies showed that changes in myopia and astigmatism were not statistically significant [19], while others found that changes in myopia drift and astigmatism generally returned to the preoperative level within 3–12 months of follow-up, and some studies described continuous changes [5–8]. Based on the structural and physiological basis of the retina, the visual acuity of ordinary people can reach 2.0 or better, but in clinical practice, the visual acuity of more than 1.5 or better is very rare, and the main factor is the aberration caused by the visual system, which will reduce the quality of retinal imaging and limit the vision. HOAs, being an optical defect, cannot be corrected by any reliable method at present. The integrity of tear film can affect higher-order aberration, and the unsmooth anterior corneal surface induced by tear film destruction is the key factor leading to the increase of HOAs [20–24]. Pupil diameter changes [25–28] and adjustments [29–33] can also cause changes in HOAs. Therefore, we used fixed targets in our instruments and designed to control the patient's pupil diameter to eliminate the effect on HOAs measurement. In order to reduce the instability factors of tear film, all of our surgeries were performed with a perinatal incision. Studies have shown that this incision has less influence on the tear film after surgery than the limbal incision [34, 35]. In addition, in order to eliminate the interference of time factor after blink on tear film stability, the measurement time of wavefront aberration after each blink was controlled within 10 seconds. HOAs play an important role in visual quality, but there are few reports on the effect of horizontal rectus surgery on HOAs. Most of the studies are short-term studies on postoperative aberration, mainly concentrated within 1 week after surgery, and the longest study is 3 months after surgery. Seo [36] et al. studied the changes of HOAs after external rectus retromigration surgery and found that HOAs briefly increased 1 week after surgery and returned to baseline level 1 month later. Bae [37] et al. investigated the changes in corneal HOAs after bilateral lateral rectus posterior migration and unilateral lateral rectus posterior migration combined with medial rectus resection and found that, in the lateral rectus posterior migration group, there were significant changes in *Z*_4_^0^ and *Z*_4_^2^ at 1 week postoperatively compared to preoperatively and *Z*_4_^4^at 1 month; in the unilateral lateral rectus posterior migration combined with internal rectus resection group, there were statistically significant changes in *Z*_4_^−4^ only at 1 month and 3 months postoperatively. This suggests that the effect of horizontal rectus surgery on HOAs appears to be transient and reversible.

In our study, all subjects underwent unilateral lateral rectus recession combined with a medial rectus resection. Instead of studying only the 5 mm pupil diameter, this study also investigated the whole-eye aberration at a 3 mm pupil diameter to simulate a large pupil in a dark environment and a small pupil in a bright environment. The whole-eye aberration includes anterior surface aberration and intraocular aberration. The posterior surface of the cornea, atrial aqueous, lens, and vitreous humor together influence intraocular aberration, among which the lens is dominant. HOAs, spherical aberration, coma, trefoil, and secondary astigmatism all play important roles in the formation of higher-order aberrations and retinal imaging, respectively. The effect of the aberration on the central axis on visual quality is much greater than that of the peripheral aberration. For example, compared with trefoil (*Z*_3_^−3^, *Z*_3_^3^), coma (*Z*_3_^−1^, *Z*_3_^1^) was more likely to affect visual quality than trefoil (*Z*_3_^−3^, *Z*_3_^3^). As the order increases, the aberration decreases; that is to say, its influence on the overall visual quality decreases. Therefore, our study analyzes the aberration to the fourth order.

Under 3 mm pupil diameter, LOAs increased one week after surgery and returned to the preoperative level one month after surgery. Under 5 mm pupil diameter, total aberration and LOAs increased one week after surgery and recovered to the preoperative level one month after surgery. Strabismus causes a transient and reversible myopic drift. There are also parts of the present study that differ from previous studies, which rarely go to six months. We found that total aberration, LOAs, defocus, HOAs, and secondary astigmatism increased at 6 months postoperatively at 3 mm pupil diameter; total aberration and LOAs increased at 3 months and 6 months postoperatively at 5 mm pupil diameter, and defocus and astigmatism increased at 6 months postoperatively. It is speculated that this has little to do with surgery and may be related to the growth of the eye axis in adolescents, as there was not much change in the older adults in our study. Early aberration change factors may lie in the effect of surgery on corneal curvature. The cornea is a transparent tissue that maintains its elasticity through the interaction of intraocular pressure, extraocular muscles, and eyelids. Altering the attachment position of the extraocular muscles and/or changing the contact area between the muscles and the eye may alter the biomechanics of the cornea. In addition, the healing process of eyelid congestion and edema, scleral incisions, etc. can also cause different degrees of morphological changes in the cornea. We generally believe that most of the aberration changes are due to corneal astigmatism, but there should be other causes of the effect, such as the lens. The truncation, displacement, and suturing of the extraocular tendons during surgery can interfere with the blood supply to the ciliary body, which in turn causes dysfunctional blood circulation to the ciliary body, thereby altering the curvature of the lens and increasing the astigmatism of the eye. Therefore, changes in aberration after horizontal strabismus surgery may be related to impaired ciliary blood circulation in addition to corneal curvature. Axial length is the preferred indicator for monitoring myopia progression [38]. Lee et al. [5] found that axial length increased significantly in strabismus patients 1 day after surgery but returned to preoperative levels within 1 month after surgery and remained stable during follow-up. The increase in axial length at 3 and 6 months postoperatively in our study may explain the increase in LOAs in patients at 6 months postoperatively, an outcome that may be less related to surgery and more related to the patient's myopia progression, suggesting further prevention and control of myopia. Whether the change in strabismus in some patients 6 months after surgery, especially in patients with intermittent exotropia, is correlated with myopia needs further study. Whether long-term changes in aberration are completely unrelated to strabismus surgery requires further follow-up and more studies to prove.

We did not observe changes in postoperative HOAs, coma, spherical aberrations, trefoil, or secondary astigmatism. Lau et al. [39] confirmed that the larger positive spherical aberration and *Z*_3_^−3^ were, the smaller *Z*_3_^3^ was, and the slower ocular axis growth was. Hiroka et al. [40] also showed a similar trend of spherical aberration and ocular axis growth but observed only a significant association with coma, but no significant association with trefoil. From these observational studies, we conclude that HOAs are related to the eye axis and that an increase in the eye axis may be a compensatory way to reduce higher-order aberrations and promote myopia development. In much the same way as previous studies, our findings show that horizontal rectus surgery did not have a long-term effect on HOAs. Although we added for the first time a comparison of whole-eye aberrations between the 3 mm and 5 mm pupil diameter conditions, this was also not statistically significant and a larger sample size is needed to refine the study.

In this study, whole-eye aberration was investigated, which consists of anterior corneal surface aberration and intraocular aberration. The amputation, displacement, and suturing of the extraocular muscle in the process of surgery will block the blood supply of the ciliary body, thereby causing the blood circulation disorder of the ciliary body, changing the curvature of the lens, increasing the divergence of the eyeball, and even the possible impact on aberration. In addition, this study covered a wide range of ages, and lens changes may have a greater impact on aberration. Although the partial results of total eye aberration in our study were negative, the study lasted longer and was more comprehensive than previous studies at home and abroad that only observed anterior corneal surface aberration and were mostly limited to 3 months. This can provide a more accurate reference for clinical work and provide a better idea and basis for further research.

Our study may have some shortcomings. First, due to the epidemic and other factors, the follow-up loss rate is high, and the sample size is not as good as expected. Second, we chose time points from 7 days to 6 months postoperatively. Due to the high number of daytime patients and poor patient compliance with the 1-day postoperative examination, we did not investigate data from the early postoperative time (1–3 days postoperatively) and the high rate of loss at 1-year follow-up due to the epidemic, data were not included in the statistics, and further clinical studies are needed to elucidate preoperative changes and long-term prognosis.

## 4. Conclusion

In conclusion, we found that horizontal rectus surgery had a transient effect on LOAs and almost no effect on HOAs. Long-term follow-up is recommended after strabismus surgery to observe eye position and binocular visual function. Because of the high prevalence of strabismus in adolescents, long-term observation of the eye axis and aberration is recommended.

## Figures and Tables

**Table 1:** Comparison of RMS of preoperative and postoperative aberrations at 3 mm pupil diameter (mean ± SD, *μ*m).

	Preoperative	1 week	1 month	3 months	6 months	*P* value^*∗*^
RMS of total aberration	0.84 ± 0.41	0.94 ± 0.40	0.91 ± 0.39	0.99 ± 0.34	1.05 ± 0.40	<0.01
RMS of LOAs	0.83 ± 0.41	0.94 ± 0.41	0.91 ± 0.40	0.98 ± 0.34	1.04 ± 0.41	<0.01
Defocus	0.76 ± 0.45	0.87 ± 0.43	0.80 ± 0.48	0.91 ± 0.36	0.95 ± 0.47	<0.01
Astigmatism	0.24 ± 0.18	0.29 ± 0.21	0.28 ± 0.20	0.28 ± 0.20	0.30 ± 0.18	0.085
RMS of HOAs	0.06 ± 0.02	0.08 ± 0.04	0.07 ± 0.03	0.07 ± 0.04	0.08 ± 0.03	0.013
Coma	0.04 ± 0.02	0.05 ± 0.03	0.05 ± 0.02	0.04 ± 0.04	0.05 ± 0.03	0.157
Spherical aberration	0.01 ± 0.02	0.01 ± 0.02	0.01 ± 0.02	0.01 ± 0.02	0.01 ± 0.02	0.571
Secondary astigmatism	0.01 ± 0.01	0.02 ± 0.01	0.02 ± 0.01	0.01 ± 0.01	0.02 ± 0.01	0.001
Trefoil	0.04 ± 0.02	0.05 ± 0.03	0.04 ± 0.03	0.04 ± 0.03	0.04 ± 0.03	0.086

**Table 2:** Comparison of RMS of preoperative and postoperative aberrations at 5 mm pupil diameter (mean ± SD, *μ*m).

	Preoperative	1 week	1 month	3 months	6 months	*P* value^*∗*^
RMS of total aberration	2.44 ± 1.20	2.75 ± 1.19	2.57 ± 1.27	2.88 ± 1.01	3.01 ± 1.12	<0.01
RMS of LOAs	2.42 ± 1.21	2.73 ± 1.20	2.55 ± 1.13	2.87 ± 1.01	2.99 ± 1.13	<0.01
Defocus	2.24 ± 1.29	2.54 ± 1.25	2.29 ± 1.39	2.68 ± 1.05	2.70 ± 1.34	0.002
Astigmatism	0.66 ± 0.51	0.76 ± 0.56	0.79 ± 0.54	0.80 ± 0.58	0.92 ± 0.56	0.02
RMS of HOAs	0.24 ± 0.08	0.28 ± 0.13	0.26 ± 0.09	0.25 ± 0.11	0.28 ± 0.12	0.308
Coma	0.15 ± 0.09	0.15 ± 0.08	0.17 ± 0.10	0.16 ± 0.12	0.18 ± 0.11	0.451
Spherical aberration	0.05 ± 0.07	0.04 ± 0.09	0.06 ± 0.08	0.04 ± 0.08	0.05 ± 0.07	0.542
Secondary astigmatism	0.04 ± 0.02	0.07 ± 0.06	0.06 ± 0.03	0.05 ± 0.03	0.06 ± 0.04	0.108
Trefoil	0.11 ± 0.06	0.13 ± 0.09	0.11 ± 0.07	0.12 ± 0.06	0.12 ± 0.08	0.425

**Table 3:** Comparison of axial length before and after operation (mean ± SD, mm).

	Preoperative	1 week	1 month	3 months	6 months	*P* value^*∗*^
AL	24.26 ± 1.06	24.28 ± 1.08	24.29 ± 1.08	24.34 ± 1.07	24.52 ± 1.10	<0.01

## Data Availability

Data are available from the corresponding author upon reasonable request.
